# Role of integrin α4 in the inhibition of fibrosis in activated hepatic stellate cells by *Periplaneta americana* extract

**DOI:** 10.3389/fphar.2025.1517491

**Published:** 2025-03-04

**Authors:** Ying Fang, Ye Liu, Dingchun Li, Yi Miu, Kexuan Chen, Jv Zhou, Lijuan Xie, Xinting Chen, Jingyan Wu, Ying Zhu, Lechun Lv, Wu Li

**Affiliations:** ^1^ Department of Infectious Disease, The First Affiliated Hospital of Kunming Medical University, Yunnan, China; ^2^ Yunnan Key Laboratory of Stem Cell and Regenerative Medicine, School of Rehabilitation, Kunming Medical University, Yunnan, China

**Keywords:** *Periplaneta americana* extract (PAE), ITGA4, LX2, HSC-T6, fibrosis

## Abstract

This study aims to investigate the role of integrin α4 (ITGA4) in the inhibition of hepatic stellate cells (HSCs) fibrosis by *Periplaneta americana* extract (PAE), as well as to explore its molecular mechanisms. *In vitro* experiments utilized TGFβ-induced LX2 and HSC-T6 cells to examine the anti-fibrotic effects of PAE, particularly through ITGA4 overexpression, to elucidate its involvement in PAE-mediated inhibition via the PI3K-AKT signaling pathway. Cell viability was assessed using the CCK-8 method, and the IC_50_ for PAE was determined through statistical analysis. We evaluated cell proliferation using scratch and EDU assays, and migration capabilities using Transwell assays. Molecular mechanisms were investigated through western blot (WB), quantitative PCR (QPCR), and transcriptome analysis. Results indicate that PAE reduces hepatic fibrosis by curbing hepatic stellate cells (HSCs) proliferation, migration, collagen synthesis, inflammatory cytokine production, and epithelial-mesenchymal transition (EMT). Additionally, while PAE suppressed ITGA4’s high expression in activated HSCs, ITGA4 overexpression counteracted PAE’s effects on HSC proliferation, migration, and collagen synthesis. These findings demonstrate that PAE primarily mitigates fibrosis in activated HSCs by inhibiting ITGA4, thus delivering anti-fibrotic effects in the liver.

## 1 Introduction

Liver fibrosis (LF) involves the excessive deposition of extracellular matrix (ECM) and fibrotic scarring, predominantly generated by activated myofibroblasts. These myofibroblasts evolve from hepatic stellate cells (HSCs), bone marrow-derived stem cells, and epithelial or endothelial cells via epithelial-mesenchymal transition (EMT) or endothelial-MT (EndoMT) (Iwaisako et al., 2014; Higashi et al., 2017; Kisseleva and Brenner, 2021). HSCs are pivotal in ECM activation. In China, where liver disease is prevalent, fibrosis often progresses to cirrhosis and potentially to liver cancer (Yin et al., 2013). To date, no definitive cure exists for LF, prompting significant research focus on inhibiting HSCs activation to forestall the condition (Mannaerts et al., 2015; Arroyo et al., 2021; Yan et al., 2021). Therefore, inhibition of hepatic stellate cell activation and prevention of hepatic fibrosis significantly limit the development of liver diseases.

The use of traditional Chinese medicine in treating Liver fibrosis (LF) has garnered significant attention (Hu et al., 2017; Tee et al., 2018; Du et al., 2020). PAE exhibits a broad spectrum of biological activities and therapeutic effects, notably in chronic heart failure (CHF) (Lu et al., 2018), gastrointestinal ulcers (Li et al., 2020), wound healing (Liao et al., 2023; Yang et al., 2024), organ fibrosis (Liu et al., 2018; Lv et al., 2023), and cancer (Ma H. et al., 2022). Regarding organ fibrosis, PAE has been demonstrated to inhibit hepatic fibrosis in experimental animals, though few *in vitro* studies have been conducted, and the specific action mechanism remains unclear (Li et al., 2018).

Recent studies have demonstrated that integrin inhibition can alleviate Liver fibrosis (Rahman et al., 2022). Integrin α4 (ITGA4) has been implicated in various disorders, including gastrointestinal diseases (Dotan et al., 2020), cancer (Xie et al., 2020), among others, though specific research on ITGA4 in LF remains sparse. Our previous work established that ITGA4 mediates the inhibitory effects of *Periplaneta americana* extract (PAE) on carbon tetrachloride-induced hepatic fibrosis in rats (Tao et al., 2022). revealed that EP3-mediated NK cell activation protects against injury-induced hepatic fibrosis via the PKC/Spic/Itga4 signaling pathway in mice, and (Schonfeld et al., 2023) discovered that arginine methylation of ITGA4 prevents alcohol-related liver disease in mice by obstructing fibrosis development. These findings indicate a correlation between ITGA4 and liver fibrosis. This study delves into ITGA4’s role in the PAE-mediated attenuation of hepatic fibrosis in TGFβ-stimulated LX2 and HSC-T6 cells to elucidate the interaction between ITGA4 and the suppression of LF by PAE.

## 2 Materials and methods

### 2.1 Drugs

PAE was obtained from Ganlong capsules, produced by Saino Pharmaceuticals (Kunming Saino Pharmaceutical Co., Ltd., State Pharmaceutical Approval No. Z20050113). The capsules contain a brownish powder, with PAE as the primary component. Dissolve the powder in ddH_2_O using ultrasonic solubilization and store it protected from light. Prepare a master batch at a final concentration of 30 mg/mL and store at 4°C.

### 2.2 Cell culture

HSC-T6 and LX2 cells were grown in DMEM with 10% FBS (ExCell, FSP500) and 1% penicillin-streptomycin (Gibco, 15140122) at 37°C in a 5% CO_2_ environment. The cells were purchased from Procell in Wuhan, China. After the cells reached logarithmic growth, they were co-treated for 48 h with different doses of PAE and TGFβ (5 ng/mL, MCE, China).

### 2.3 CCK-8

After 24 h of incubation, cells were transferred to 96-well plates. There was a control group, a TGFβ group with a concentration of 5 ng/mL, and PAE groups with concentrations ranging from 0.3125 mg/mL to 20 mg/mL (0.3125 mg/mL, 0.625 mg/mL, 1.25 mg/mL, 2.5 mg/mL, 5 mg/mL, 10 mg/mL, 20 mg/mL), spread throughout seven different levels. Every PAE treatment group received 5 ng/mL of TGFβ. Following 48 h, 110 µL of basal medium and CCK-8 reagent (TargetMol, America) were added to each well in a 10:1 ratio to replace the old medium. The OD was assessed at 450 nm with an enzyme counter following a 2-h incubation period. Each group was independently repeated at least three times. Inhibitory concentration values (IC_20_, IC_40_, IC_50_) for the CCK-8 assay were derived using SPSS 18.0 and expressed as mean ± standard deviation (SD).

### 2.4 EdU incorporation assay

An *in vitro* kit, Cell-light™ EdU Apollo567, was used to measure cell proliferation (RiboBio, China). The cells were cultured in a Petri dish, exposed to PAE and TGFβ for a duration of 48 h, stained with the EDU kit, and then photographed using fluorescence microscopy. Using ImageJ software, the cells that tested positive were analyzed. EdU incorporation assays were conducted on three independent biological replicates.

### 2.5 Transwell migration assay

1 × 10^5^ cells/mL were inoculated into Transwell inserts (100 µL per well). After 48 h, the inserts were transferred to wells containing 1 mL of 4% paraformaldehyde for fixation. The contents of the upper chamber were drained, and 200 µL of 4% paraformaldehyde was added, which was left for 30 min. Afterward, the inserts were placed in wells containing 500 µL of crystal violet staining solution for 15 min. Transwell migration assays were performed on three independent biological replicates, with the inserts subsequently examined under a microscope and photographed.

### 2.6 Wound healing assay

At approximately 80% confluence, a 20 µL pipette tip was used to create a horizontal scratch. After sterilising the cells three times with PBS, the media was changed to fresh serum-free medium. The migration distance was measured by taking images at 0, 12, and 24 h and examining them under a microscope. Wound healing assays were conducted on three independent biological replicates.

### 2.7 Western blot (WB)

Antibodies employed included ITGA4 (Bioss, bs-0641R), α-SMA (Proteintech, 14395-1-AP), COL1 (Proteintech, 14695-1-AP), TNFα (Affinity, AF7014), IL-6 (Affinity, DF 6087), ECA (Bioss, bs-10009R), NCA (Bioss, bs-1172R), Snail (CST, 3879T), Vim (CST, 5741S), TGF-β1 (Abcam, ab92486), FAK (CST, 71433S), PI3K (Abcam, ab191606), AKT (CST, 4691L), P-FAK (CST, 3284), P-PI3K (Abcam, ab278545), P-AKT (CST, 4060s), β-actin (Proteintech, 60008-1-Ig), and GAPDH (Proteintech, 60004-1-lg). Western blot analyses were conducted using at least three independent biological replicates.

### 2.8 RNA-sequencing

Two groups of LX2 cells were analyzed: a TGFβ-treated group (5 ng/mL) and a TGFβ + PAE-treated group (6.120 mg/mL), with three samples in each group, designated as TGF21-23 and TGF31-33, respectively. All data have been uploaded to NCBI with a BioProject accession number of PRJNA1177452 (https://www.ncbi.nlm.nih.gov/bioproject/PRJNA1177452 100).

### 2.9 Total RNA extraction and quantitative real-time PCR

Total RNA was extracted from LX2 and HSC-T6 cells using TRIzol reagent (Thermo Fisher, 15596018). cDNA was synthesized using the FastKing RT Kit (with gDNase). Gene-specific mRNA sequences were retrieved from PubMed, and primers were designed based on the CDS sequences using Beacon Designer 7.90. qRT-PCR analyses were performed with at least three independent biological replicates. The primer sequences included: β-actin (F: 5′-GCA​GGA​GTA​CGA​TGA​GTC​CG-3′; R: 5′-ACG​CAG​CTC​AGT​AAC​AGT​CC-3′); α-SMA (F: 5′-TCA​AGA​TTA​TTG​CTC​CTC-3’; R: 5′-CTG​GAA​GGT​AGA​TAG​AGA-3′); COLⅠ (F: 5′-AAG​AAG​ACA​TCC​CTG​AAG-3′; R: 5′-AGA​TAC​AGA​TCA​AGC​ATA​CA-3′); ITGA4 (F: 5′-TTG​AAG​ATA​TTG​CTA​TTG​G-3′; R: 5′-GAG​TAT​GTA​GAG​GAG​ATG-3′).

### 2.10 Overexpressing α4 integrin

Genomeditech vectors (Shanghai, China) were used to achieve ITGA4 overexpression. Thermo Fisher, China’s Lipofectamine™ 3000 was used to transfect cells with ITGA4 plasmids. The ITGA4 plasmid was transfected for 24 h, followed by a treatment with TGFβ and PAE for an additional 48 h. Additional primer sequences for ITGA4 were GAPDH (F: 5′-TTG​CCC​TCA​ACG​ACC​ACT​TT-3′; R: 5′-TGG​TCC​AGG​GGT​CTT​ACT​CC-3′); ITGA4 (F: 5′-ATC​TCG​TCA​AC-CTT​CTC​A-3′; R: 5′-CAT​CTA​CAT​AGC​CAT​TAT​TAT​CTG-3′).

### 2.11 Statistical methods

Data analysis was performed using GraphPad Prism 9.0. Results from three independent, repeated experiments are presented as mean ± standard deviation (SD). Statistical differences among various groups were determined through one-way ANOVA, with a threshold of P < 0.05 for statistical significance.

## 3 Results

### 3.1 PAE inhibits the migration and proliferation of LX2 and HSC-T6 cells

**FIGURE 1:**
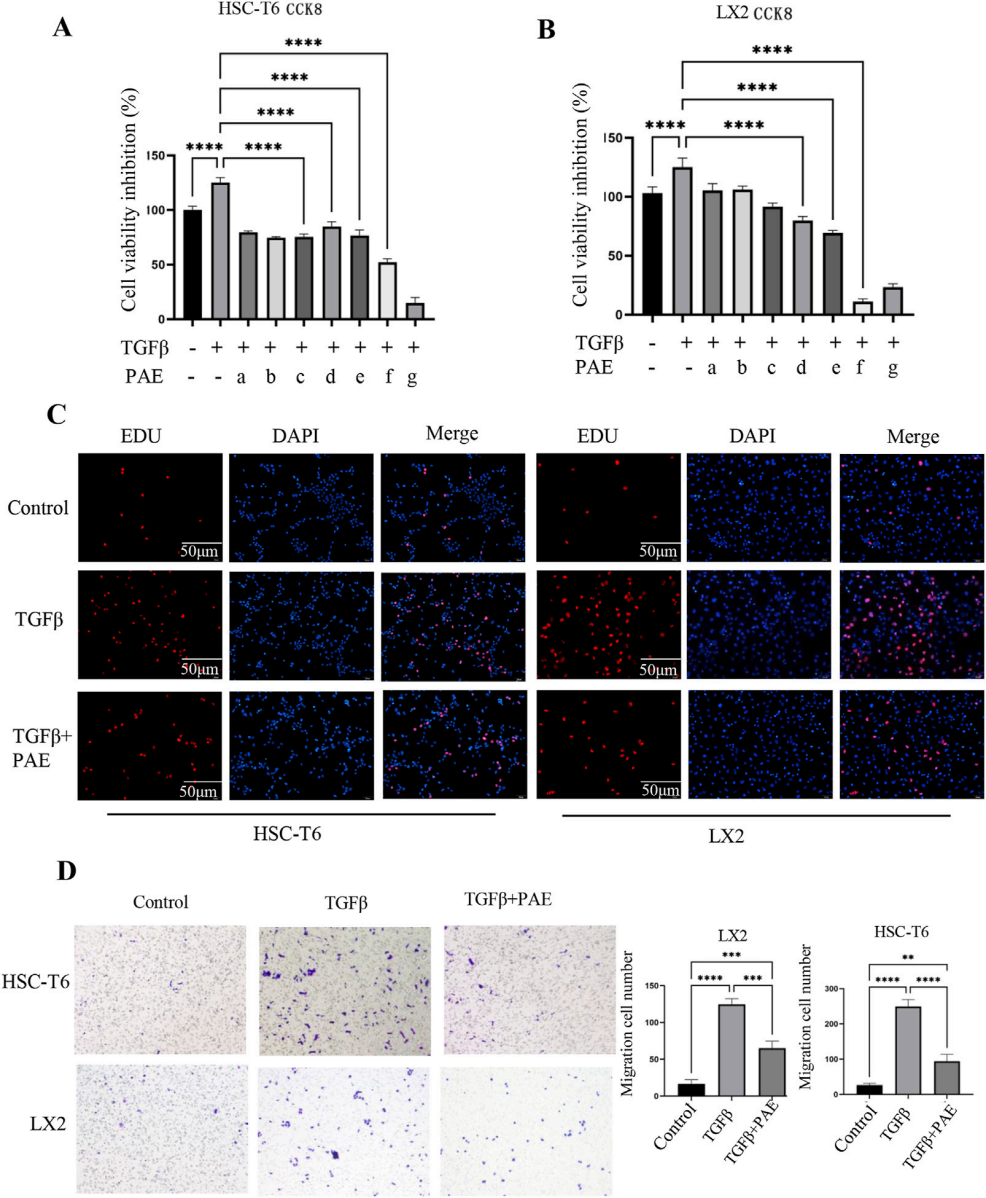
Effect of PAE on TGFβ-induced cell viability and migration in HSC-T6 and LX2 cells. **(A, B)** show the inhibition of cell viability and IC_50_ values of HSC-T6 and LX2 cells from the CCK8 assay. The PAE concentrations ranged from 0.3125 mg/mL to 20 mg/mL (a–g: 0.3125 mg/mL, 0.625 mg/mL, 1.25 mg/mL, 2.5 mg/mL, 5 mg/mL, 10 mg/mL, 20 mg/mL, n = 5). **(C)** EDU assay results for HSC-T6 and LX2 cells. **(D)** Transwell migration assay of HSC-T6 and LX2 cells; Where TGFβ was administered at a concentration of 5 ng/mL, and PAE was applied at the IC_50_ concentration for both LX2 and HSC-T6 cells. *p < 0.05, **p < 0 0.01, ***p < 0.001.

The effect of PAE on TGFβ-induced activation of HSCs were evaluated by measuring the viability of LX2 and HSC-T6 cells through the CCK8 assay. The data demonstrated that TGFβ at 5 ng/mL significantly increased the activation of HSC (Figures 1A, B). Conversely, PAE effectively decreased cell proliferation in a concentration-dependent manner. Statistically, the IC_20_, IC_40_, and IC_50_ values for PAE were 2.29 ± 0.33 mg/mL, 4.53 ± 0.38 mg/mL, and 6.02 ± 0.34 mg/mL in LX2 cells, and 0.79 ± 0.10 mg/mL, 4.27 ± 0.34 mg/mL, and 8.89 ± 1.26 mg/mL in HSC-T6 cells, with the most significant effects observed at the IC_50_ concentration for both cell types. The subsequent experiments were thus designed around these concentrations (IC_20_, IC_40_, and IC_50_).

The Transwell assay demonstrated that PAE reduced the migration of activated HSC (Figure 1D). TGFβ increased the migration of LX2 and HSC-T6 cells, whereas PAE markedly reduced the number of migrating cells from these cell lines. In the Edu assay, it was observed that PAE inhibited the proliferation of HSCs stimulated by TGFβ (Figure 1C), indicating that PAE attenuates LF by suppressing both cell migration and proliferation.

### 3.2 RNA sequence analysis identifies genes and signaling pathways regulated by PAE

**FIGURE 2:**
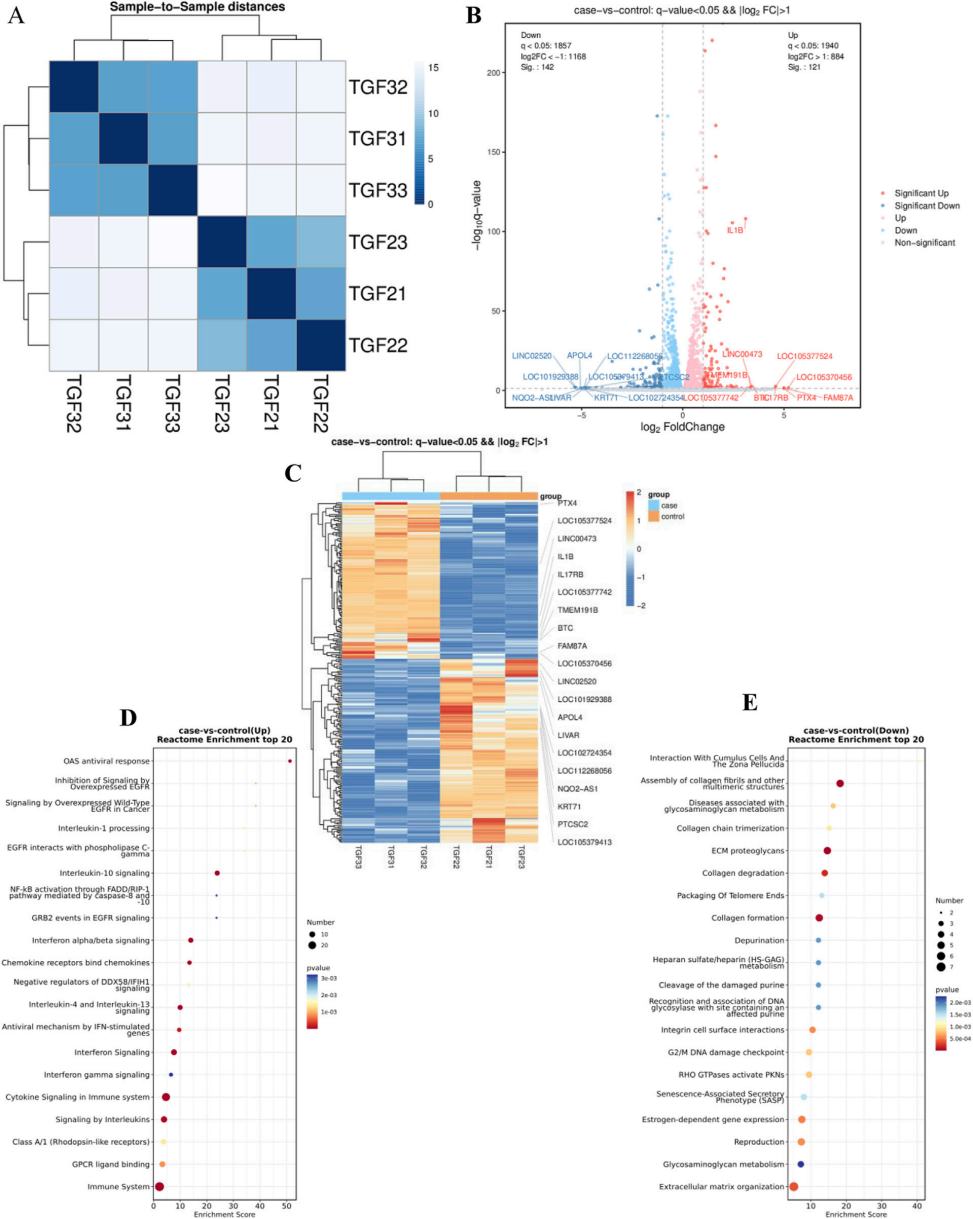
RNA sequencing analysis of several components: **(A)** Correlation analysis of RNA-seq data. **(B)** Visualization of differential RNA expression data using EnhancedVolcano. **(C)** Heatmap illustrations depicting differentially expressed genes (DEGs) in samples treated with TGFβ alone (TGF21-23) and those treated with TGFβ plus PAE (TGF31-33), n = 3. **(D)** and **(E)** Reactome pathway analysis identified pathways associated with downregulated **(D)** and upregulated **(E)** genes in the RNA-Seq data. The control group was treated with TGFβ, while the experimental group received TGFβ plus PAE.

RNA sequencing was conducted to ascertain the effects of PAE on activated HSCs (LX2), with three independent biological replicates. Correlation analysis confirmed that replicates clustered distinctly for each sample group (Figure 2A). Post-quality control, differential expression was determined, with significant upregulation and downregulation noted at an FDR < 0.05 and |log2 (FC)| > 0.5. This analysis identified 142 upregulated and 121 downregulated genes (Figures 2B, C).

Enrichment analysis using the Reactome database highlighted that downregulated genes were predominantly involved in the assembly of collagen fibrils (including COL1A2; COL5A3; COL9A2; LOXL4; MMP13), ECM proteoglycans (including BGN; COL1A2; COL5A3; COL9A2; LUM), collagen degradation (including COL1A2; COL5A3; COL9A2; LOXL4; MMP13), and collagen formation (COL1A2; COL5A3; COL9A2; MMP13), among other related processes (Figure 2D). Conversely, upregulated genes primarily participated in cytokine signaling, interleukin-10 signaling, and interferon α/β signaling within the immune system (Figure 2F).

The transcriptomic results revealed that genes associated with collagen fibers and inflammatory pathways were substantially downregulated under PAE’s influence, aligning with our observation that PAE mitigates hepatic fibrosis. Notably, ITGA4 was among the downregulated genes, corroborating previous findings from our group regarding its role in PAE-mediated inhibition of carbon tetrachloride-induced LF in rats. The precise mechanism by which ITGA4 contributes to PAE’s inhibition of LF warrants further investigation.

### 3.3 PAE inhibits hepatic fibrosis gene expression and EMT-Related index expression in LX2/HSC-t6 cells

**FIGURE 3:**
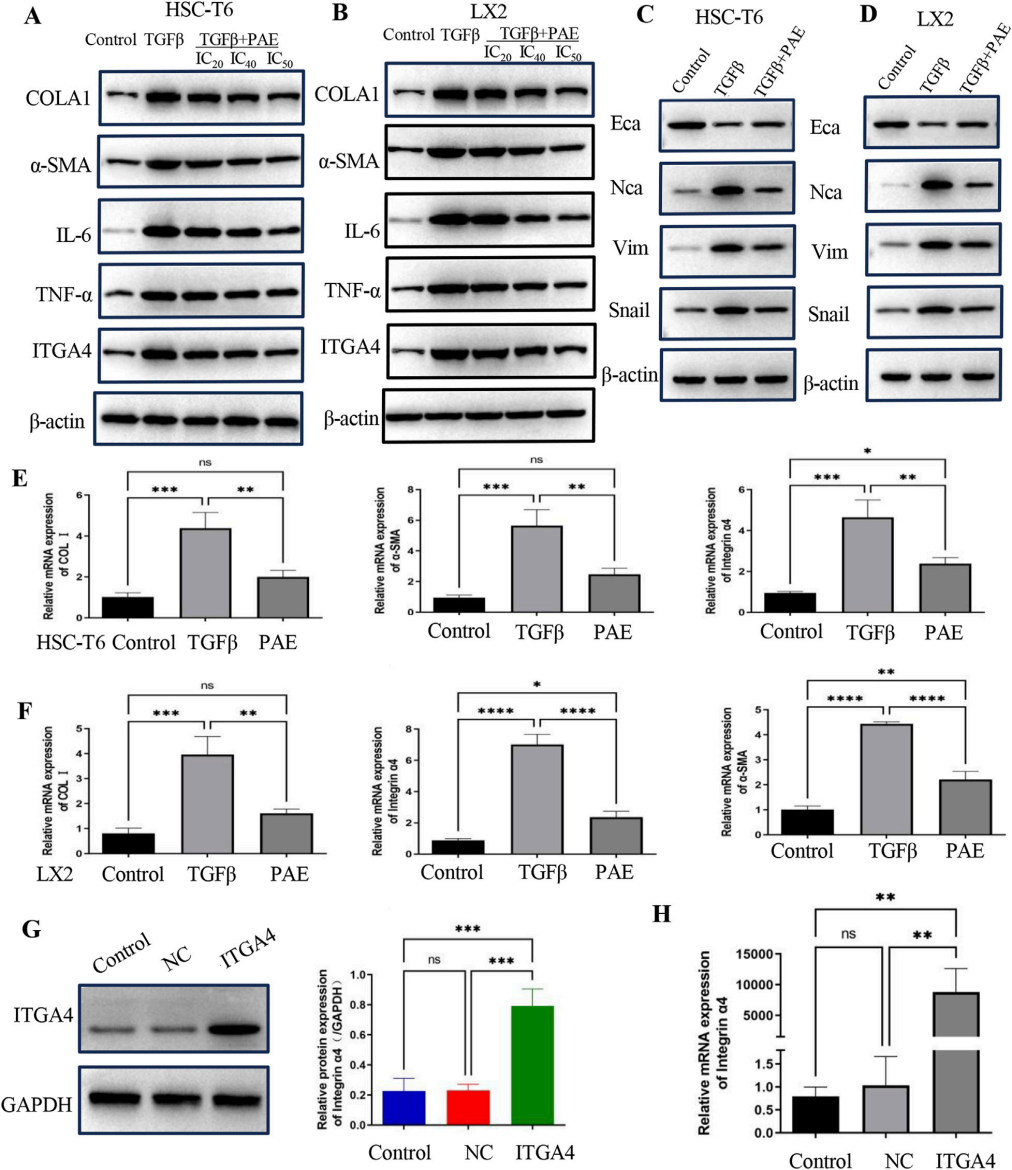
PAE inhibited the expression of LF and EMT-related genes in LX2 and HSC-T6 cells. **(A, B)** Western blot analysis measured protein levels associated with LF genes in both LX2 and HSC-T6 cells. **(C, D)** EMT protein levels were assessed in LX2 and HSC-T6 cells. **(E, F)** mRNA levels of LF genes were quantified in LX2 and HSC-T6 cells, respectively. **(G)** Protein levels following overexpression of the ITGA4 plasmid. **(H)** mRNA levels following ITGA4 plasmid overexpression were also evaluated. The blank control group, empty plasmid control (NC), and ITGA4 overexpression group were included for comparison. Statistical significance was indicated as *p < 0.05, **p < 0 0.01, ***p < 0.001.

To further explore the anti-fibrotic effect of PAE on activated HSCs, both WB and QPCR analyses were employed. These analyses revealed elevated levels of α-SMA and COL1 proteins and mRNAs in TGFβ-stimulated LX2 and HSC-T6 cells compared to controls, signifying the activation by TGFβ (Figures 3A–D). Treatment with PAE led to a reduction in α-SMA and COL1 at both the transcriptional and translational levels (Figures 3A–D), with the most notable decrease observed at the IC_50_ concentration of PAE. Additionally, PAE also lowered the expression of inflammatory markers TNFα and IL-6 in these cells (Figures 3A, B). Furthermore, while ITGA4 was highly expressed in these cells, PAE significantly decreased its expression (Figures 3A–D).

Further WB experiments assessed changes in EMT indicators. Expression of NCA, Snail, and Vim increased under TGFβ treatment compared to controls, while PAE inhibited their expression (Figures 3E, F). These results suggest PAE’s anti-fibrotic action through the inhibition of collagen, inflammatory factors, and EMT expression. Importantly, PAE was found to reduce the high expression of ITGA4 in TGFβ-induced HSCs.

Using an overexpressed ITGA4 plasmid, the effects on LF and its interaction with PAE’s inhibitory action were explored. Further investigation into the role of ITGA4 in LF was conducted through QPCR and WB. These assays confirmed an increase in ITGA4 expression at both mRNA and protein levels in LX2 cells following transfection with the ITGA4 plasmid, in contrast to cells transfected with an empty vector or the untreated group (Figures 3G, H).

### 3.4 ITGA4 enhances TGFβ-Induced LX2 cell viability and migration

**FIGURE 4:**
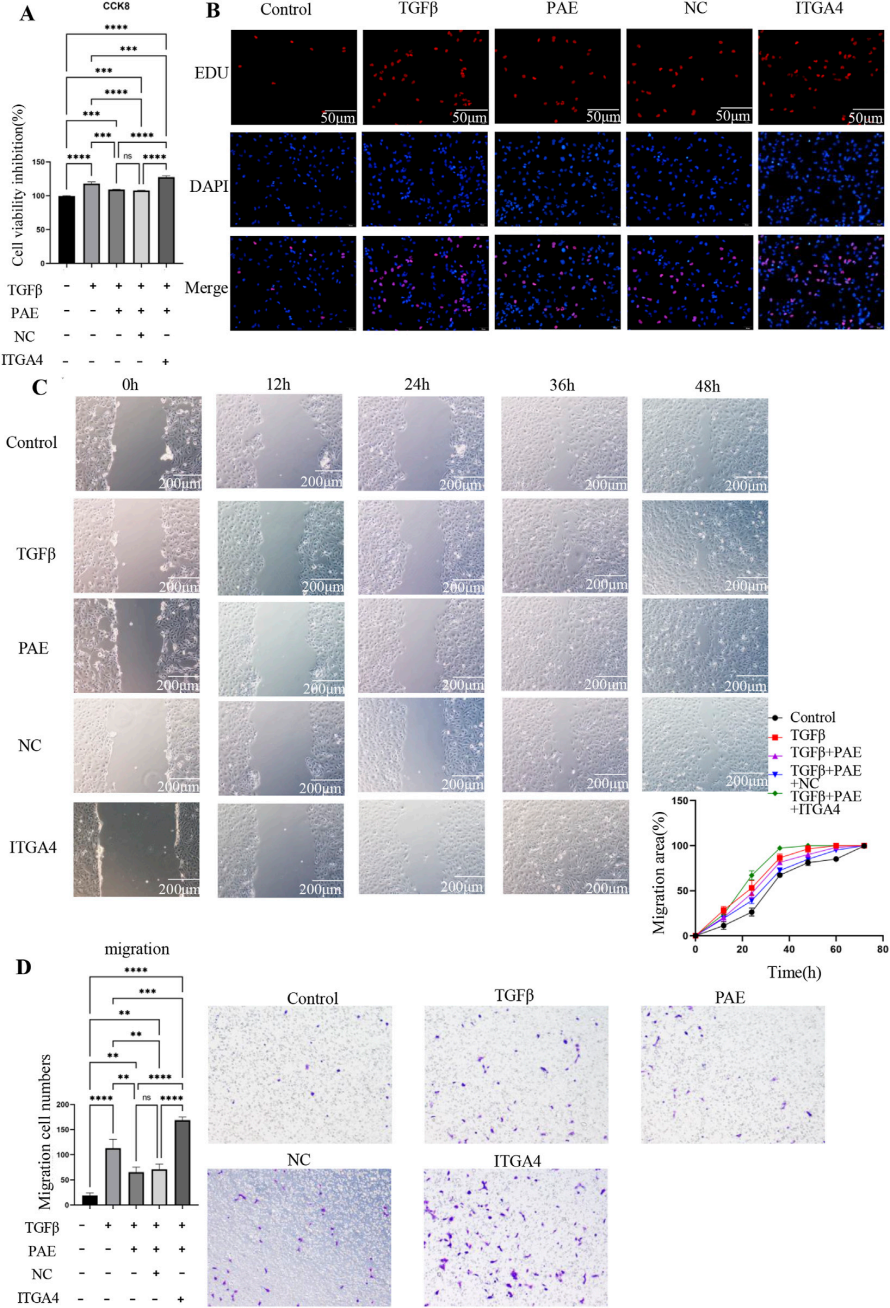
ITGA4 promotes the proliferation and migration of activated HSCs. **(A)** CCK8 assay results. **(B)** EDU assay results following overexpression of the ITGA4 plasmid. **(C)** Wounding assay results. **(D)** Transwell migration assay results with ITGA4 plasmid overexpression. Control: blank control group, TGFβ, TGFβ-induced activation group, PAE, TGFβ + PAE. NC: TGFβ + PAE + NC, ITGA4: TGFβ + PAE + ITGA4. Statistical significance was indicated as *p < 0.05, **p < 0 0.01, ***p < 0.001.

The impact of ITGA4 on LX2 cell proliferation was evaluated using CCK8 and Edu assays. CCK8 results indicated that ITGA4 increased cell viability and promoted proliferation compared with the empty plasmid control (NC) group (Figure 4A). Furthermore, ITGA4 enhanced LX2 cell viability more than TGFβ treatment alone, suggesting its significant role in enhancing cell proliferation (Figure 4A). This was also supported by the Edu assay, where ITGA4 significantly increased the proliferation of activated LX2 cells, counteracting PAE’s inhibitory effect (Figure 4B). Additionally, both the Transwell migration and scratch assays showed that ITGA4 enhanced the migration of activated LX2 cells and reversed PAE’s inhibitory effects on their migration (Figures 4C, D). Compared with the TGFβ group, ITGA4 significantly promoted LX2 cell migration, indicating that the ability of ITGA4 to promote migration not only reversed the inhibitory effect of PAE but also exceeded the ability of TGFβ to promote cell activation. This highlights ITGA4’s substantial role in promoting the proliferation and migratory abilities of LX2 cells.

### 3.5 ITGA4 reverses PAE inhibition of hepatic fibrosis

**FIGURE 5:**
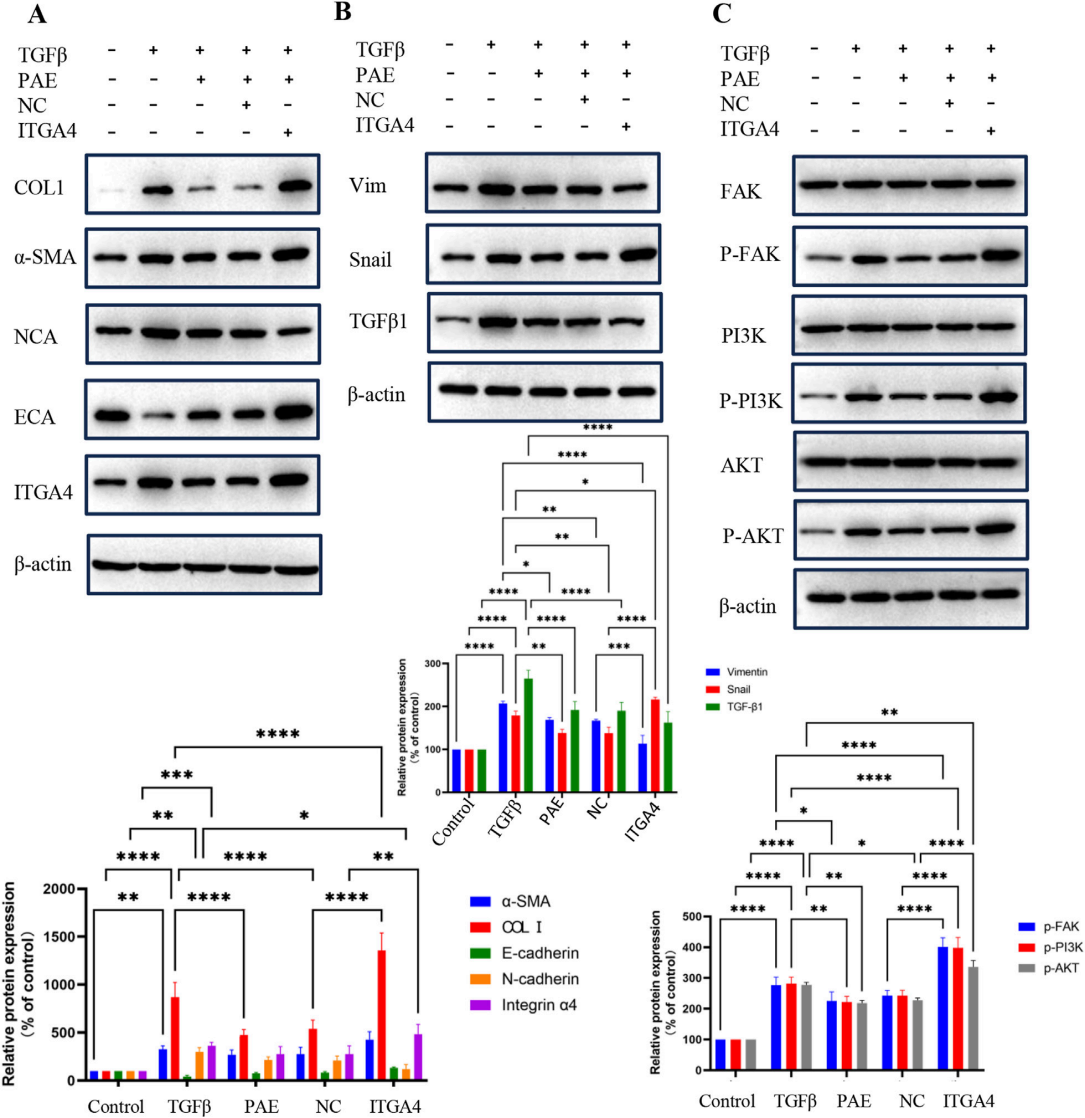
PAE inhibits hepatic fibrosis by suppressing ITGA4. **(A, B)** Protein expression levels of ITGA4 reversing the inhibitory effects of PAE in HSCs. **(C)** Probing PAE inhibition of ITGA4 in the PI3K-AKT signaling pathway. Statistical significance was indicated as *p < 0.05, **p < 0 0.01, ***p < 0.001.

The study also included an assessment of α-SMA, COL1, and EMT markers via the WB assay to elucidate the regulatory role of ITGA4 in the pathogenesis of LF. The findings revealed that in TGFβ-treated HSCs, overexpression of ITGA4 reversed PAE’s inhibition of fibrosis. Specifically, in PAE-treated cells, ITGA4 promoted the expression of α-SMA, COL1, ECA, and Snail, while it inhibited the expression of NCA and Vim (Figures 5A, B). Although both PAE and ITGA4 suppressed TGF-β1 expression, the difference between their effects was not statistically significant (Figure 5B). The promotional effect of ITGA4 on these markers counteracted the inhibitory impact of PAE, underscoring ITGA4’s role in exacerbating hepatic fibrosis. These results further support that ITGA4 counteracts the inhibitory effect of PAE on hepatic fibrosis, highlighting ITGA4’s role in promoting this condition.

### 3.6 PAE inhibits LF in LX2 cells through the ITGA4/FAK/PI3K/AKT pathway

Transcriptomic analyses indicated that PAE’s inhibition of hepatic fibrosis involved the ECM pathway (Figure 2E). To validate this, the PI3K-AKT pathway was examined. Results showed that PAE suppressed the expression and phosphorylation of FAK, PI3K, and AKT in TGFβ-activated LX2 cells, whereas ITGA4 overexpression enhanced these expressions and phosphorylations. However, the expression levels of FAK, PI3K, and AKT were comparable across groups (Figure 5C). These findings suggest that PAE’s inhibition of LF in LX2 cells occurs through the ITGA4/FAK/PI3K/AKT pathway, emphasizing the critical role of ITGA4 inhibition in treating LF.

## 4 Discussion

In the present study, we explored the association between ITGA4 expression and the inhibitory actions of PAE on LF. Prior studies have shown elevated ITGA4 levels in rat models of LF induced by carbon tetrachloride. To elucidate the mechanistic link between ITGA4 and PAE’s inhibition of hepatic fibrosis, *in vitro* experiments were conducted on TGFβ-induced LX2 and HSC-T6 cells. ITGA4 was found to significantly influence the inhibition of fibrogenesis and progression in HSC by PAE. In normal hepatocytes, ITGA4 was either lowly expressed or absent but was highly expressed in damaged cells. Various studies have highlighted ITGA4’s critical role in tissue repair, tumorigenesis, and development (Li et al., 2022; Ryu et al., 2024). Liver fibrosis is a pathological condition characterized by chronic and sustained damage to HSCs, potentially progressing to cirrhosis and hepatocellular carcinoma. PAE can mitigate hepatic fibrosis, with most previous studies concentrating on its ability to suppress collagen expression and the associated TGFβ signaling pathway. In our research, we utilized Ganlong capsules—primarily composed of PAE—for the first time on TGFβ-induced HSCs for a transcriptome analysis. This approach clearly demonstrated that PAE not only inhibits hepatic fibrosis but also reduces hepatic stellate cell proliferation, migration, collagen expression, and EMT expression, thus effectively preventing liver fibrosis.

While the specific relationship between ITGA4 and liver fibrosis remains unclear, there is extensive research on the integrin family in this context. Integrins such as α8β1 (Nishimichi et al., 2021), αvβ1 (Han et al., 2021), αvβ6 (Peng et al., 2016) have been shown to mitigate hepatic fibrosis through negative regulatory effects, while α4β7 (Rai et al., 2020) and α5 (Ma Y. et al., 2022) appear to inhibit fibrosis via positive effects. Thus, integrins are potential targets for treating liver fibrosis (LF). ITGA4 is primarily associated with cellular proliferation, migration, invasion, and the progression to cirrhosis and hepatocellular carcinoma. This research delves deeper into the potential involvement of ITGA4 in hepatic fibrosis and examines the suppressive effects of PAE on HSC. Notably, it proposes for the first time that ITGA4 might contribute to the mechanism by which PAE mitigates LF.

Building on our earlier findings, we observed that ITGA4 expression was significantly elevated in cases of hepatic fibrosis prompted by carbon tetrachloride in rats, but this expression was notably decreased following treatment with PAE. This study further explores the relationship between ITGA4 and PAE’s inhibition of hepatic fibrosis. The effects of PAE were predominantly demonstrated through its suppression of hepatic fibrosis in experimental animals. Using TGFβ-induced HSCs, we investigated PAE’s inhibitory effects on hepatic fibrosis and the role of ITGA4 in this process. PAE was found to inhibit hepatic fibrosis by reducing activated HSC proliferation, migration, and the expression of collagen fiber genes COL1 and α-Sma, as well as inflammatory markers TNFα and IL-6, and EMT markers (ECA, NCA, Vim, Snail). PAE also suppressed ITGA4 expression in TGFβ-induced HSC, which naturally expresses high levels of ITGA4. Conversely, ITGA4 overexpression reversed PAE’s suppression of proliferation, migration, and collagen fiber gene expression. Meanwhile, we found that ITGA4 had a potent pro-fibrotic effect. ITGA4 could promote liver fibrosis by promoting hepatic stellate cell value-addition, migration, and collagen expression. This suggests PAE may inhibit liver fibrosis primarily through suppressing ITGA4.

However, this study did not explore whether ITGA4 could reverse the inhibition of inflammatory factor expression in hepatic fibrosis by PAE. Literature indicates that the ITGA4 family plays a role in leukocyte recruitment during injury, infection, and inflammation, with applications in treatments for inflammatory bowel disease (Dotan et al., 2020; Tyler et al., 2022) and lupus nephritis (Ryu et al., 2024), and protection against non-alcoholic steatohepatitis (Rai et al., 2020). We hypothesize that ITGA4 is also involved in the PAE-mediated inhibition of inflammatory responses in liver fibrosis (LF). While TGFβ is a key factor in LF, not all integrin-related LF is induced by TGFβ, indicating that ITGA4’s role in promoting LF may not depend solely on TGFβ. Transcriptomic and WB analyses showed that PAE’s suppression of ITGA4 expression in hepatic fibroblasts was linked to the ECM pathway, and overexpressed ITGA4 promoted P-FAK/P-PI3K/P-AKT expression, counteracting PAE’s inhibitory effects. This suggests that PAE inhibits hepatic fibrosis through the ITGA4/FAK/PI3K/AKT signaling pathway, independent of the TGFβ-mediated pathway. Previous *in vivo* experiments demonstrated high ITGA4 expression solely in liver-injured tissues, necessitating additional *in vivo* experiments for further validation.

Additionally, continuous animal experiments could not be conducted; instead, reliance was placed on the prior work of our research group to undertake *in vitro* experimental explorations. The relationship between ITGA4 and PAE might also pertain to other liver diseases, necessitating further exploration.

In conclusion, our findings demonstrate ITGA4’s role in PAE-mediated inhibition of hepatic fibrosis, positioning ITGA4 as a potential target for preventing hepatic fibrosis. PAE inhibits HSC fibrosis predominantly through the ITGA4/FAK/PI3K/AKT signaling pathway and suppresses EMT expression by inhibiting ITGA4. However, the lack of supportive *in vitro* experiments in this study calls for further research to confirm that PAE inhibits LF by targeting ITGA4.

## Data Availability

The datasets presented in this study can be found in online repositories. The names of the repository/repositories and accession number(s) can be found in the article/supplementary material.
